# A Hyper-Chaotically Encrypted Robust Digital Image Watermarking Method with Large Capacity Using Compress Sensing on a Hybrid Domain

**DOI:** 10.3390/e24101486

**Published:** 2022-10-18

**Authors:** Zhen Yang, Qingwei Sun, Yunliang Qi, Shouliang Li, Fengyuan Ren

**Affiliations:** 1 School of Information Science and Engineering, Lanzhou University, Lanzhou 730000, China; 2 School of Electronic and Information Engineering, Lanzhou Jiaotong University, Lanzhou 730070, China; 3 Tsinghua National Laboratory for Information Science and Technology, Tsinghua University, Beijing 100084, China

**Keywords:** DWT, SVD, hyper-chaotic map, digital image watermark, compressive sensing, information hidden

## Abstract

The digital watermarking technique is a quite promising technique for both image copyright protection and secure transmission. However, many existing techniques are not as one might have expected for robustness and capacity simultaneously. In this paper, we propose a robust semi-blind image watermarking scheme with a high capacity. Firstly, we perform a discrete wavelet transformation (DWT) transformation on the carrier image. Then, the watermark images are compressed via a compressive sampling technique for saving storage space. Thirdly, a Combination of One and Two-Dimensional Chaotic Map based on the Tent and Logistic map (TL-COTDCM) is used to scramble the compressed watermark image with high security and dramatically reduce the false positive problem (FPP). Finally, a singular value decomposition (SVD) component is used to embed into the decomposed carrier image to finish the embedding process. With this scheme, eight 256×256 grayscale watermark images are perfectly embedded into a 512×512 carrier image, the capacity of which is eight times over that of the existing watermark techniques on average. The scheme has been tested through several common attacks on high strength, and the experiment results show the superiority of our method via the two most used evaluation indicators, normalized correlation coefficient (NCC) values and the peak signal-to-noise ratio (PSNR). Our method outperforms the state-of-the-art in the aspects of robustness, security, and capacity of digital watermarking, which exhibits great potential in multimedia application in the immediate future.

## 1. Introduction

The excessive spread of Internet is a double-edged sword. On the one hand, information accessing and sharing are more convenient than ever. On the other hand, illegal data copying, reproduction, and editing are more rampant as well [1]. From social media to government online management, from telemedicine to cloud services, from commercial activities to military, all the important information storage and communication which heavily relies on the Internet are prone to be collected and undermined by unpermitted malicious attackers. The protection, certification, and authentication of the Information Sovereignty are particularly urgent.

Digital watermark embedding is one of the promising solutions for data security, which has been widely used in various scenes. By embedding specific relevant information into the host data, the digital watermarking scheme could be used as an information hiding means to ensure the reliability and origination of online data transformation, so that the multimedia data are protected and secured [2]. The categories of digital watermark can be classified as the visible and imperceptible one according to its visibility. The former often suffers from insufficient robustness, and does not constitute a proof of ownership either [3]. For practical reasons, the latter are preferred by academic researchers [4].

Imperceptible watermarks involve the spatial domain one and the transform domain one referring to distinctions of embedding methods [5]. In the spatial domain, watermarks are embedded via modifying the pixel values of the original image. Harahap et al. [6] proposed a spatial domain watermarking scheme, that in which pictures or text in binary form are embedded into the host data. They manage to embed a 150×150 grayscale watermark into a 518×649 color image, and pursue low time cost on the embedding process. Although most spatial domain watermarking schemes have less computational complexity than the transform domain schemes to perform, their anti-attack characteristics is far poorer than the latter which has a higher robustness on average [7].

In the transform domain, watermarks are first embedded by changing the frequency coefficients of image transformed mainly by discrete wavelet transform (DWT), discrete cosine transform (DCT) or Contourlet Transform. Then, the embedded coefficients are inverse-processed to form the watermarked image. Thus, the watermark often owns better invisibility and stronger robustness to some common image-processing operations and attacks [8]. Vaidya and Mouli [9] proposed a method based on hybrid transformation with DWT, Contour transformation, Schur transformation, and SVD to embed a 62×62 gray watermark image into a 512×512×3 color image, which reveals good resistance to signal processing attacks. Kumar et al. [10] proposed an enhanced transform domain watermark scheme based on DWT, DCT, and SVD with set partitioning in a hierarchical tree (SPIHT), which can restore a 256×256 watermark from a 512×512 host image with a high resistance under various attacks. Ambadekar et al. [11] encrypted a 90×90 watermark image, and then embedded it into a 228×228 host image through DWT transformation, who claimed a high robustness of their method against various types of attacks. However, with all these schemes listed above, only a very limited size of watermark images are embedded into the host image.

The invisible watermarking techniques can be further divided into three types: blind, semi-blind, and non-blind ones according to the amount of information of the original watermark required for extracting. Non-blind methods need the original signal, whose application is limited due to the unsure access to the original signal. Blind methods require no information of the original signal at all for watermark recovering, but are full of challenges [12]. For semi-blind watermarking techniques, side information rather than the whole original watermark is needed for watermark recovery [13], which aims to make a compromise between the blind and the non-blind schemes.

Note that an imperceptible image watermark scheme should be built with large capacity, strong robustness, high security, and good imperceptibility [7]. A watermarking scheme with great capacity could surely contain more information, offering more essential and detailed materials supporting its function like providing authentication or information hidden, e.g., embedding different copyrights into images like digital collections with more than one author [14], or during the distribution of the media content from producers, retailers, and customers to protect the benefits of both the producer and the middle distributors [15]. Unfortunately, most state-of-the-art schemes as discussed above are insufficient, especially in the capacity. Therefore, putting forward algorithms for preserving the desired properties of a watermarking procedure is quite essential.

In this paper, we proposed a hyper-chaotically encrypted robust digital image watermarking scheme which has a high capacity by using compressive sensing (CS) on a hybrid domain. The main contributions of our work are summarized as follows:

(1) Capacity: CS is introduced into our scheme to compress the watermark for pursuing high capacity. Compared with those existing grayscale watermark algorithms, our scheme is eight times over them in capacity, which brings a great improvement to the watermark capacity.

(2) Robustness: The proposed scheme combining DWT with SVD has high robustness, which can effectively restore all the embedded watermarks with high NCC values when confronting with the threats of several typical strong attacks.

(3) Security: A hyperchaotic system with a broad chaos range is introduced for watermark image encryption, which can provide a huge secret key space. The proposed scheme is capable of guaranteeing the security of the watermarks in the host image. Even if the embed algorithm is cracked, the attacker would never be able to restore the encrypted watermark images validly from the host image without the correct secret keys, thus overcoming the FPP of SVD-based watermarking techniques.

The rest of our paper is organized as follows: Section 2 refers to preliminaries including a few fundamentals of CS, SVD, DWT, and the hyperchaotic system TL-COTDCM. In Section 3, the proposed scheme is elaborated in detail. In Section 4, experiment results and discussions are given. In Section 5, we present conclusions for the whole work.

## 2. Preliminaries

### 2.1. Discrete Wavelet Transform

**Figure 1:**
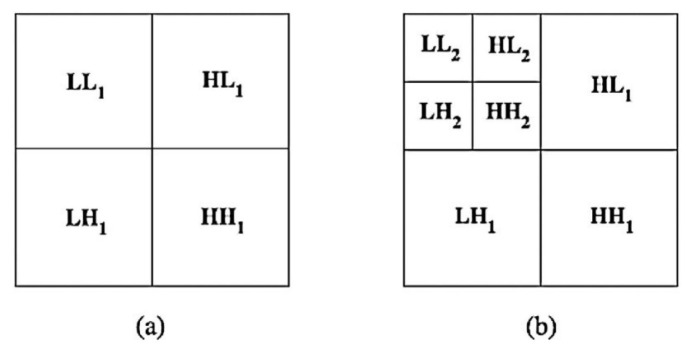
(**a**) 1-level DWT, (**b**) 2-level DWT.

DWT is used to transform an image from spatial to frequency domain, which can decompose an image into four sub-bands known as LL, LH, HL, and HH sub-bands, and provide the low resolution, the horizontal, diagonal, and vertical details of the image separately in the spatial domain [16]. Compared with the discrete cosine transform (DCT), DWT has less computational complexity and is more favorable in image processing for the utilization of informative wavelets with details from both spatial and frequency domains rather than merely frequency as in DCT. In particular, the precise spatial frequency localization of DWT allows for the exploitation of the masking effect of the human visual system (HVS) so that only the region corresponding to a modified DWT coefficient would be changed [17]. Several wavelet filters could be chosen to perform the 2D-DWT, such as Coiflets, Haar and Daubechies, etc. As shown in Figure 1, DWT could be iteratively executed on a signal to split it into low and high-frequency sub-bands level by level until a sufficient decomposition is obtained [18]. In our scheme, we choose 1-level DWT [19,20] with the Haar wavelet filter for the low computing complexity and high image resolution.

### 2.2. 2D Compressive Sensing (CS)

#### 2.2.1. Basic Principle

CS believes sparse signals, which are thought of as an incomplete description of the original signals before, could be used to reconstruct original signals [21]. It was firstly introduced by Candes and Donoho in 2006 [22]. According to the CS theory, a 2D signal *A* of size a×a could be measured by:

(1) $$\alpha =\Phi_{1}\Psi^{T}A$$

where α is the sparse coefficient, $\Phi_{1}$ is a b×a measurement matrix, and Ψ is an a×a orthogonal basis [23]. Let β be the sparse coefficient vector of α in the Ψ domain; then, β could be obtained by:

(2) $$\beta =\Psi^{T}\alpha^{T}=\Psi^{T}A^{T}\Psi \Phi_{1}^{T}=\gamma \Phi_{1}^{T}$$

where $\gamma =\Psi^{T}A^{T}\Psi$, which is a 2D Ψ transformation of signal $A^{T}$. The M×M measurement value *B* is obtained by measuring β with another measurement matrix $\Phi_{2}$:

(3) $$B=\Phi_{2}\beta =\Phi_{2}\gamma \Phi_{1}^{T}$$

where *B* is the result of the 2D CS transformation.

The original signal *A* could be recovered from the measurement value *B* by solving the following convex optimization problem:

(4) $$\begin{array}{c} \delta ={\arg min||\gamma ||}_{0},\;s.t.B=\Phi_{2}\gamma \Phi_{1}^{T} \end{array}$$

The CS theory enables the signal storage to consume quite a lot less memory, which also can be viewed as a symmetric cryptosystem with the original signal, the measurement matrix and measurement value corresponding to the plaintext, secret key and ciphertext, respectively [24]. It performs sampling, compression, and encryption simultaneously possessing a wide range of application scenarios, such as image compression and data encryption.

#### 2.2.2. The TVAL3 Algorithm

There have been many effective reconstruction algorithms such as the orthogonal matching pursuit (OMP) [25], subspace pursuit (SP) [26], and stochastic gradient pursuit (SGP) [27] to pursue a favorable reconstruction quality. In our scheme, we apply the TVAL3 algorithm for compressive sensing implementation.

The TVAL3 algorithm developed by Li [28] uses the total variation (TV) regularization to solve the CS problems, which improves the recovered image visual quality by preserving the edges or boundaries more accurately. It has been proved that this algorithm could recover the original signal within an affordable running time for noise-free images with various of measurement matrices [28].

### 2.3. The Hyperchaotic System TL-COTDCM

A hyperchaotic system is defined as chaos with more than one positive Lyapunov exponent, which indicated that the dynamics of the system are expanded in more than one direction and give rise to a more complex attractor. The enhanced behaviors of the hyperchaotic system offer it a wider application in nonlinear circuits, secure communications, lasers, and synchronizations [29].

**Figure 2:**
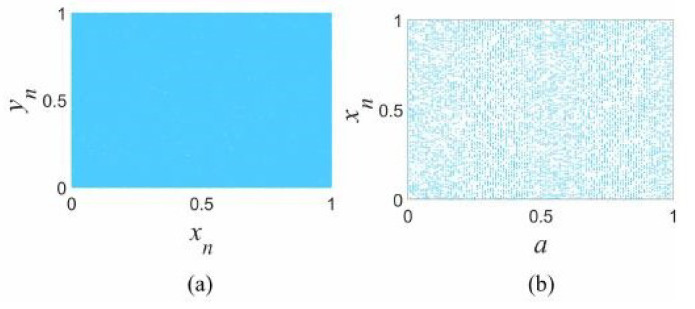
(**a**) phase diagram, (**b**) bifurcation diagrams of the TL-COTDCM system.

The TL-COTDCM we proposed in our previous work [30] has two large positive Lyapunov exponents, indicating a quite complex dynamics behavior, which is defined as follows:

(5) $$\left(x_{n+1},y_{n+1}\right)=\left\{\begin{array}{c} (4bx_{n}\left(1-x_{n}\right)+dy_{n}^{2})\;\mathrm{mod}\;1\\ \left\{\begin{array}{c} (\frac{4cy_{n}\left(1-y_{n}/a\right)}{a}+ex^{2})\;\mathrm{mod}\;1\\ if\;0\leq y_{n}<a\\ (\frac{4c\left(1-y_{n}\right)\left(1-\left(1-y_{n}\right)/\left(1-a\right)\right)}{a}+ex^{2})\;\mathrm{mod}\;1\\ if\;a\leq y_{n}\leq 1 \end{array}\right. \end{array}\right.$$

where *a*, *b*, *c*, *d*, and *e* are the control parameters, and a is defined in the range [0,1]. We set *a* = 0.5, *b* = 4, *c* = 4, *d* = 2, and *e* = 2, the phase diagram and bifurcation of TL-COTDCM is shown in Figure 2. It is obvious that the phase diagram of this system is able to fill the whole 2D space, indicating the complex nonlinear dynamics characteristics of TL-COTDCM.

In this paper, the initial values of state variables are set as $\left(x_{0},y_{0}\right)=\left(0.6,0.98\right)$, which is used to yield measurement matrix for compressive sensing as well as secret keys for sampled image encryption.

### 2.4. SVD

SVD, a common linear algebra tool which is widely utilized in signal processing, artificial intelligence and image compression, etc. One merit of applying SVD is that the singular values of an image vary minimally when it is marginally disturbed [31].

An image could be viewed as a matrix, an array of nonnegative scalar, from the point of view of linear algebra [32]. Assuming that *A* is an N×N grayscale image, then the SVD of it is depicted as

(6) $$A=USV^{T}$$

where both *U* and *V* are N×N orthogonal matrices and *S* is an N×N diagonal matrix containing singular values of the original image matrix *A*.

SVD is widely used in watermarking algorithms as it adapts to different rectangular size images. The watermarks would be embedded effectively on an SVD executed host image.

## 3. Methods

In this section, we present our watermark scheme in detail. The proposed watermarking scheme is composed of four main steps: compression and encryption, embedding, extracting, and decryption and decompression process. The first two procedures are performed at the sending site, while the other two are done at the receiving site after obtaining the watermarked image from the public channel. Each procedure is elaborated in detail as follows.

### 3.1. Compression and Encryption

To maximize utilization of the capacity of host image, we perform CS on original watermark images to compress them into smaller sizes for embedding. Here, we use a sampling rate ρ of 0.25 for a trade off between image quality and compression ratio, which means that only 25% of the information of the original watermark images are used for embedding. Then, the compressed watermarks are encrypted by using the order of a sorted TL-COTDCM sequence again to achieve a higher level of security. Two initial values are defined as secret keys for the TL-COTDCM to generate the chaotic sequence.

**Figure 3:**
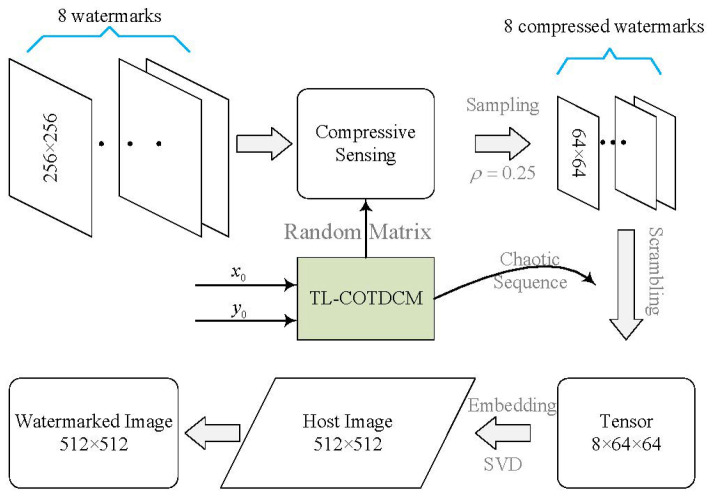
Flowchart of the watermarking method for grayscale images.

The flowchart of this procedure is shown in Figure 3. More details are described as

1. Perform compressive sampling using measurement matrix with the compression ratio ρ of 0.25 on eight grayscale watermark images (256×256) to obtain the sampled image $P^{\prime }$. Reshape $P^{\prime }$ with the size of [1, 8 ×64×64];
2. Initialize the control parameters of TL-COTDCM, input two states $x_{0}$ and $y_{0}$ to actuate the hyperchaotic system;
3. Generate chaotic sequence $U=[u_{1},u_{2},\ldots ,u_{8\times 64\times 64+800}]$ with the length of (8×64×64+800);
4. Omit the first 800 elements of *U* to avoid transient effect in the later scrambling procedure. Reorder the rest elements of *U* in ascending order, and record the position of them each element in the new sequence. Define the position sequence as $L_{8\times 64\times 64}$;
5. Utilize *L* for shuffling $P^{\prime }$ and yield the scrambled sequence CI;
6. Reshape CI into a tensor composed of eight matrices of size 64×64. Then, we obtain the compressed and encrypted images;
7. Transform the aforementioned watermarks by
  (7) $$\left\{\begin{array}{c} x_{k}\left(i,j\right)=CI_{k}\left(i,j\right)\;\mathrm{mod}\;\alpha \\ xx_{k}\left(i,j\right)=\left\lfloor \frac{CI_{k}\left(i,j\right)}{\alpha }\right\rfloor \end{array}\right.$$
  where $x_{k}\left(i,j\right)$, $xx_{k}\left(i,j\right)$ and $CI_{k}\left(i,j\right)$ are the elements located at the ith row and jth column in the kth matrix of tensor *x*, xx, and CI, respectively. 1≤i≤64, 1≤j≤64, 1≤k≤8. α is a retaining factor (RF) for controlling the embedding accuracy, a greater value of which may bring better recovery quality but induce worse imperception. It is empirically set as 7 in our schemes. $\left\lfloor \cdot \right\rfloor$ denotes round down operation.

### 3.2. Watermarks Embedding

**Figure 4:**
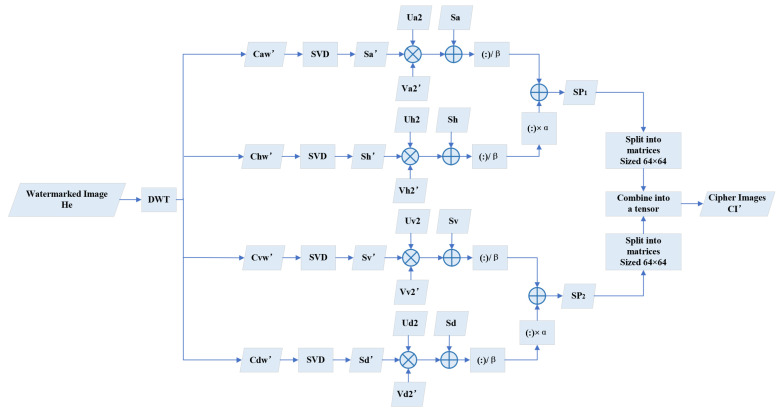
A diagram of watermarks’ embedding.

In this process, eight encrypted images are embedded into the host image to obtain a visually meaningful image. The diagram in Figure 4 displays the whole processing in detail, which can be accomplished via eight main steps.

1. To avoid the distortion of the host image caused by the probable data overflow after the embedding procedure, the pixel values of the host image are normalized in 10 to 245 according to
  (8) $$H^{\prime }=\left\lceil 10+\frac{235}{255}H\right\rceil$$
  where *H* and $H^{\prime }$ represent the original and adjusted host image, respectively. $\left\lceil \cdot \right\rceil$ denotes a round-up operation.
2. Perform 2D DWT on $H^{\prime }$
  (9) $$\left[C_{a},C_{h},C_{v},C_{d}\right]=DWT\left(H^{\prime }\right)$$
3. Perform SVD on the aforementioned DWT components via
  (10) $$U_{i}S_{i}V_{i}^{T}=C_{i}$$
  where i=a,h,v,d.
4. Generate $T_{i1}$ and $T_{i2}$ of size [256, 256] by recombining the tensor i(i=x,xx) with the following equation to prepare for embedding:
  (11) $$\begin{array}{c} T_{i1}=\left(\begin{array}{cc} i_{1} & i_{2}\\ i_{3} & i_{4} \end{array}\right)\\ T_{i2}=\left(\begin{array}{cc} i_{5} & i_{6}\\ i_{7} & i_{8} \end{array}\right) \end{array}$$
  where $i_{n}\left(n=1,2,\ldots ,8\right)$ represents the nth matrix in tensor *i*.
5. Embed $T_{i1}$ and $T_{i2}$ into $S_{i}$ obtained in Step 3 to generate $S_{i1}$ according to
  (12) $$\left\{\begin{array}{c} S_{a1}=S_{a}+\beta \cdot T_{xx_{1}}\\ S_{h1}=S_{h}+\beta \cdot T_{x_{1}}\\ S_{v1}=S_{v}+\beta \cdot T_{xx_{2}}\\ S_{d1}=S_{d}+\beta \cdot T_{x_{2}} \end{array}\right.$$
  where β is a scaling factor (SF) for embedding strength controlling. A greater β benefits anti-attack robustness but also harms imperception.
  It is empirically set as 6 in this paper.
6. Apply SVD on $S_{i1}$ once again
  (13) $$U_{i2}S_{i2}V_{i2}^{T}=S_{i1}$$
  where $U_{i}2$ and $V_{i}2$ are stored for reconstruction.
7. Calculate the embedded DWT components with U and V obtained in Step 3:
  (14) $$C_{i}w=U_{i}S_{i2}V_{i}^{T}$$
  where i=a,h,v,d.
8. Generate the watermarked host image $H_{e}$ by performing inverse discrete wavelet transformation (IDWT) on the embedded components
  (15) $$H_{e}=IDWT\left(C_{aw},C_{hw},C_{vw},C_{dw}\right)$$

### 3.3. Watermarks Extracting

**Figure 5:**
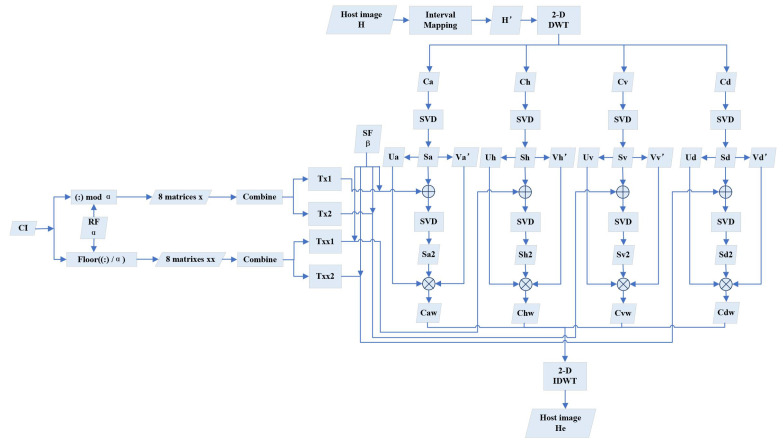
Diagram of watermark extracting.

Watermark extraction is mainly to execute an inverse operation of embedding process on the watermarked host image, which is illustrated in Figure 5. In the extracting process, we perform DWT on the watermarked image, and SVD and recombination operations are executed subsequently.

**Figure 6:**
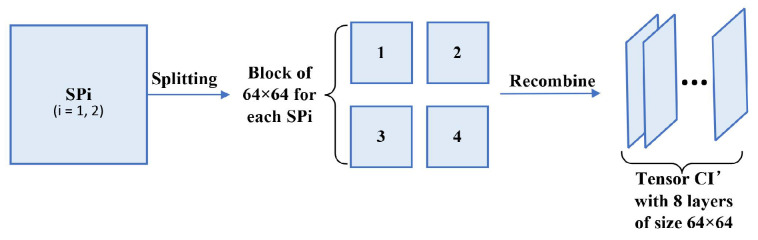
The illustration of Step 6.

1. Perform 2D DWT on the watermarked image:
  (16) $$\left[C_{aw}^{\prime },C_{hw}^{\prime },C_{vw}^{\prime },C_{dw}^{\prime }\right]=DWT\left(H_{e}\right)$$
2. Perform SVD on the obtained DWT components
  (17) $$U_{i}^{\prime }S_{i}^{\prime }V_{i}^{\prime }{}^{T}=C_{iw}^{\prime }$$
  where i=a,h,v,d.
3. Calculate the new approximate coefficient matrices $C_{newi}$, where i=a,h,v,d
  (18) $$C_{new_{i}}=U_{i2}\times S_{i}^{\prime }\times V_{i2}^{T}$$
4. Calculate the scrambled coefficients
  (19) $$W_{new_{i}}=\frac{\left(C_{new_{i}}-S_{i}\right)}{\beta }$$
  where i=a,h,v,d
5. Reconstruct the scrambled images via utilizing coefficients in Step 4
  (20) $$\left\{\begin{array}{c} SP_{1}=W_{new_{a}}+\alpha \cdot W_{new_{h}}\\ SP_{2}=W_{new_{v}}+\alpha \cdot W_{new_{d}} \end{array}\right.$$
6. Split the aforementioned $SP_{1}$ and $SP_{2}$ into four matrices separately with a size of 64×64. Then, form these eight matrices into a tensor $CI^{\prime }$, which is the extracted encrypted watermark images. This step is illustrated in Figure 6.

### 3.4. Decryption and Decompression Process

**Figure 7:**
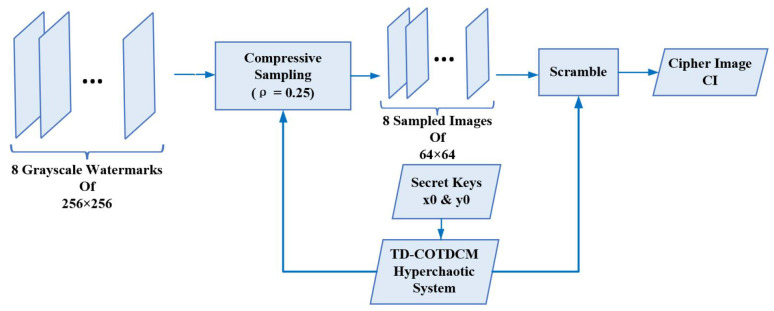
Diagram of watermark decryption and decompression.

To obtain the plain watermarks from the ciphertext obtained from extraction, first, the tensor $CI^{\prime }$ is reshaped into a vector with a size of 1 × 32,768 and scrambled subsequently by the reverse order of the hyperchaotic sequence produced by TD-COTDCM. Finally, the eight watermark images will be recovered from the decrypted tensor through CS reconstruction tools. The diagram of the process is shown in Figure 7. More descriptions are detailed as

1. Reshape the tensor $CI^{\prime }$ into a row vector with a length of 8×64×64;
2. Generate chaotic sequence $U^{\prime }$ and obtain the position sequence $L^{\prime }$ as described in Step 3 and Step 4 of part A in the Methods section; the only difference is that elements of $U^{\prime }$ are in descending order;
3. Perform the shuffle operation to $CI^{\prime }$ by using $L^{\prime }$;
4. Reshape the shuffled $CI^{\prime }$ into a tensor with eight matrices of size 64×64;
5. Recover eight grayscale watermarks of 256×256 one by one by executing the TVAL3 reconstruction tool on each block of $CI^{\prime }$ with the compression ratio ρ of 0.25.

## 4. Experimental Results and Discussion

**Figure 8:**
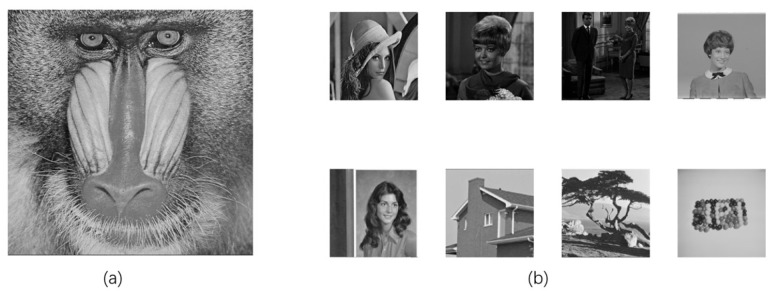
(**a**) 512×512 host image “baboon”, (**b**) eight watermark images size of 256×256.

The experiments are conducted on a PC running Windows 11 OS with x64 processor, 16 GB RAM, and 2.11 GHz CPU, and the corresponding codes are executed on the Matlab R2021a. As shown in Figure 8, the test images include the 512×512 grayscale host image “baboon” and eight grayscale watermark images with a size of 256×256, which are downloaded from the widely used public image database USC-SIPI. We compare our method with several existing recent ones which include the hybrid blind digital image watermarking methods [33], visually meaningful image encryption scheme based on a single chaotic map [18], blind color image watermarking method based on DWT and DCT [34], blind digital image watermarking based on Henon Chaotic Map and Elliptic Curve Cryptography [35], and secure and robust digital watermarking scheme based on logistic and RSA encryption [36]. The evaluated indicators involve capacity, imperceptibility, robustness, and security.

### 4.1. Capacity

To obtain an objective assessment, we apply grayscale image “baboon” uniformly to verify our proposed methods and the ones in [18,33,35,36] except for [34] require a colorful form.

**Figure 9:**
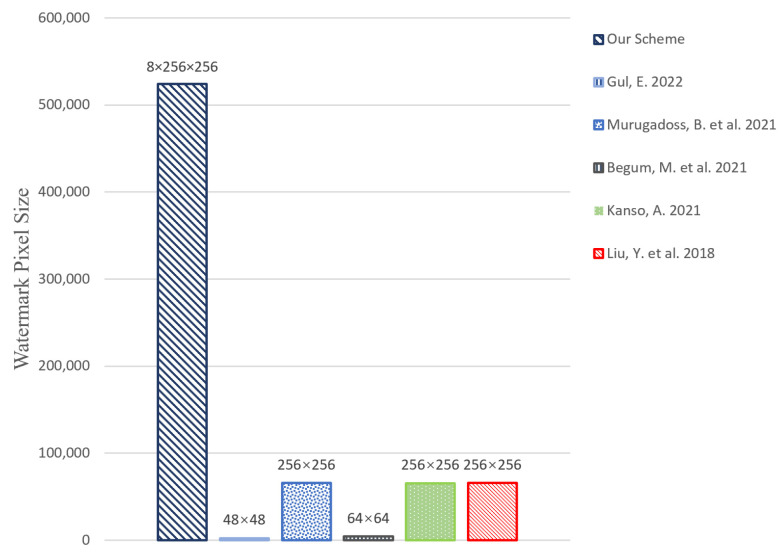
Total pixels of watermark embedded for various schemes [18,33,34,35,36].

As shown in Figure 9, our scheme ensures a total of eight watermarks with a size of 256×256 to be inserted in a host image with a size of 512×512, the works in [33,34] can embedded only 1 watermark with a size of 48×48 and 64×64, respectively. While the others [18,35,36] are one watermark with a size of 256×256. It is clear that our scheme enables maximum utilization of the capacity of the host image, which owns a higher capacity for watermarks than other schemes.

### 4.2. Robustness

Robustness refers to the resistance of an embedded watermark to attacks on the watermarked image. The normalized correlation coefficients (NCC), which can measure the similarity between the original watermark and the extracted one, is usually utilized as a criteria for robustness. NCC has a value in range 0 to 1, a greater value of which reflects a higher quality of watermark recovering. It is calculated as

(21) $$NCC=\frac{\sum_{i=1}^{n}\sum_{j=1}^{n}W\left(i,j\right)\cdot W^{\prime }\left(i,j\right)}{\sqrt{\sum_{i=1}^{n}\sum_{j=1}^{n}W{\left(i,j\right)}^{2}}\sqrt{\sum_{i=1}^{n}\sum_{j=1}^{n}W^{\prime }{\left(i,j\right)}^{2}}}$$

where *W* and $W^{\prime }$ represent the pixel value of the original watermark and the extracted one, respectively.

Note that the evaluation indicators in our experiments correspond to the mean value of eight embedded images in order to show a general performance of the proposed scheme.

#### 4.2.1. Noise Attacks

**Figure 10:**
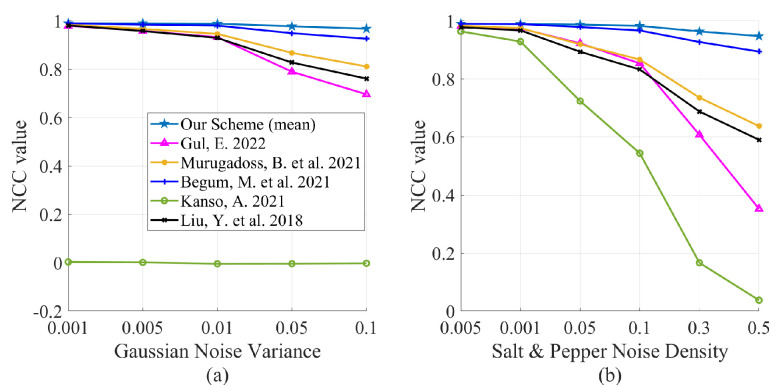
NCC values under noise attack. (**a**) Gaussian noise attack; (**b**) Salt and Pepper noise attack [18,33,34,35,36].

Two types of noise (Salt and Pepper and Gaussian noise) are selected to simulate a noise attack. The variance of Gaussian noise is set as 0.001, 0.005, 0.01, 0.05, and 0.1, while the density of Salt and Pepper noise is set as 0.005, 0.01, 0.05, 0.1, 0.3, and 0.5. NCC values for different methods under various noise attack are shown in Table 1. For Gaussian noise attack, our scheme maintains values of the NCC stably above 0.96 under different noise variance, while others show an apparent descendant tendency. In particular, the scheme in [18] almost has no resistance to Gaussian noise attack. As for Salt and Pepper noise attack, our scheme and [33] maintain NCCs more stably than others. Although the performance of [18] is a little better under this kind of attack than that under Gaussian noise attack, it still obtains the lowest score. Figure 10 also illustrates the comparison of the aforementioned watermarks schemes under different noise attacks. No matter what the attack noise type is, our scheme could resist them well.

#### 4.2.2. Geometric and JPEG Compression Attacks

**Figure 11:**
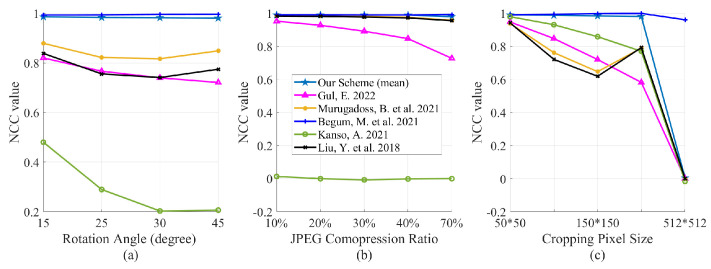
NCC values under geometric attack. (**a**) rotation attack; (**b**) JPEG compression attack; (**c**) cropping attack [18,33,34,35,36].

JPEG compression attack and Geometric attack involving rotation attack and cropping attack are used for testing watermarking schemes robust in this part. The compression ratio is set as 10%, 20%, 30%, 40%, and 70%. The rotation angle is set as $15^{\circ }$, $25^{\circ }$, $30^{\circ }$, and $45^{\circ }$; the cropping size is set as 50×50, 100×100, 150×150, and 200×200. The experimental results are shown in the latter part of Table 1. For rotation and JPEG compression attacks, the scheme in [33] and ours perform slightly better than in Ref. [35] but are obviously superior to others. As for cropping attack, we can find that the scheme in [33] and ours are still outperforming others. However, when the cropping attack of size 512×512, i.e., no information of the watermarked image is involved, the scheme in [33] can recover the watermark well, which seems unreasonable. In fact, the watermarking scheme in [33] must use the wavelet coefficients of the original watermark for extraction, but it is impossible to obtain these coefficients in the watermarked image unless the watermark is accurately extracted, i.e., one must obtain the original watermark to extract the watermark, which is contradictory. Figure 11 also shows a comparison of those watermarking schemes under different attacks. It is clear that our method performs better than others.

**Figure 12:**
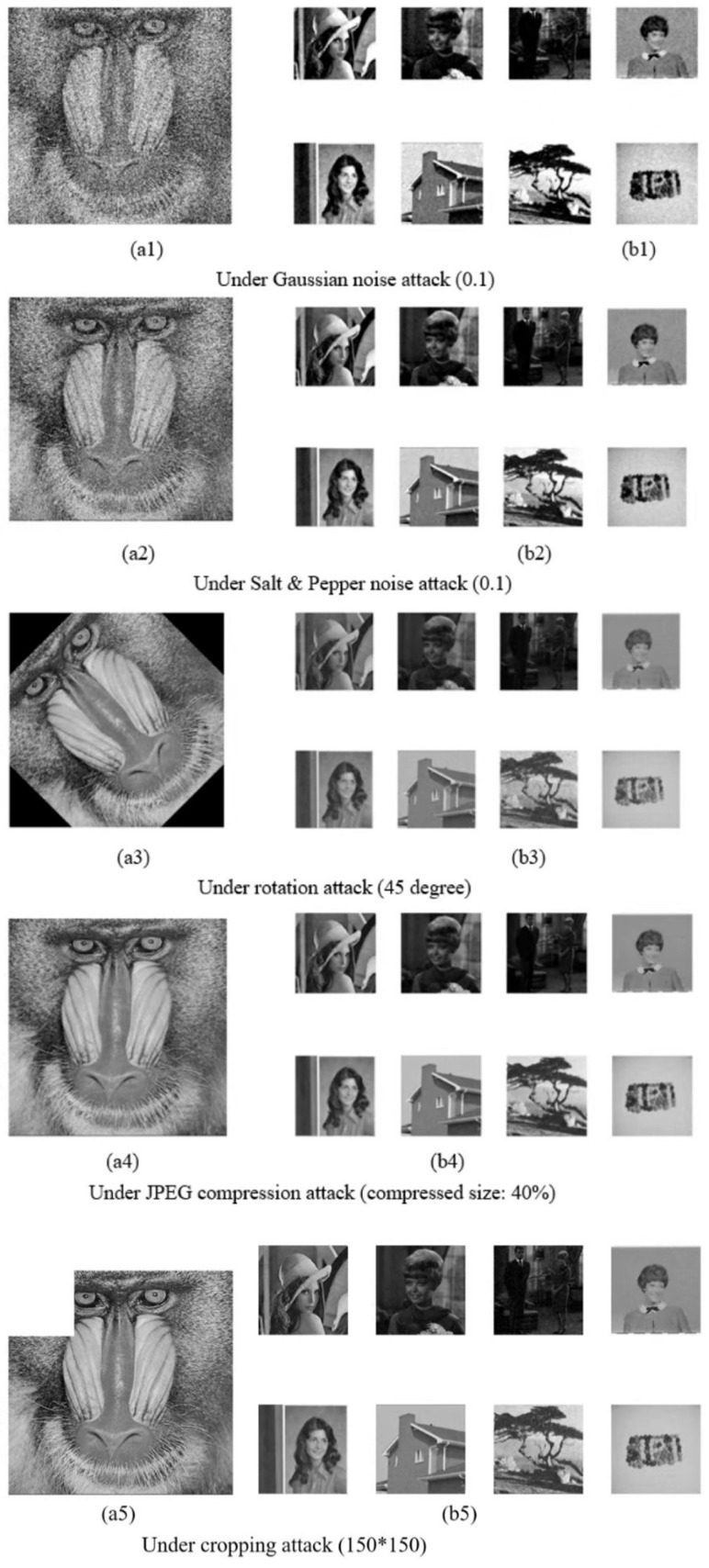
Images under various attacks. (**a1**–**a5**) denote attacked watermarked images; (**b1**–**b5**) are the extracted watermarks.

In Figure 12, we display some recovery results of our scheme against several attacks, and the recovered watermarks are still in high visual quality even with the host image under some strong attack.

### 4.3. Imperceptibility

The NCC is also used to measure the imperceptibility of watermarks in a watermarked image. Except for the aforementioned indicator, the Peak Signal-to-Noise Ratio (PSNR) measures the peak error between the watermarked host image, and the original host image is another popular indicator for imperceptibility. It is defined as

(22) $$PSNR=10log(10\times \frac{255^{2}}{MSE})$$

where MSE represents the mean square error,

(23) $$MSE=\frac{1}{M\times M}\sum_{i=1}^{M}\sum_{j=1}^{M}{\left(H\left(i,j\right)-H^{\prime }\left(i,j\right)\right)}^{2}$$

H(i,j) and $H^{\prime }\left(i,j\right)$ represent the pixel value of the original host image and the watermarked one located at the ith row and jth column, respectively.

**Figure 13:**
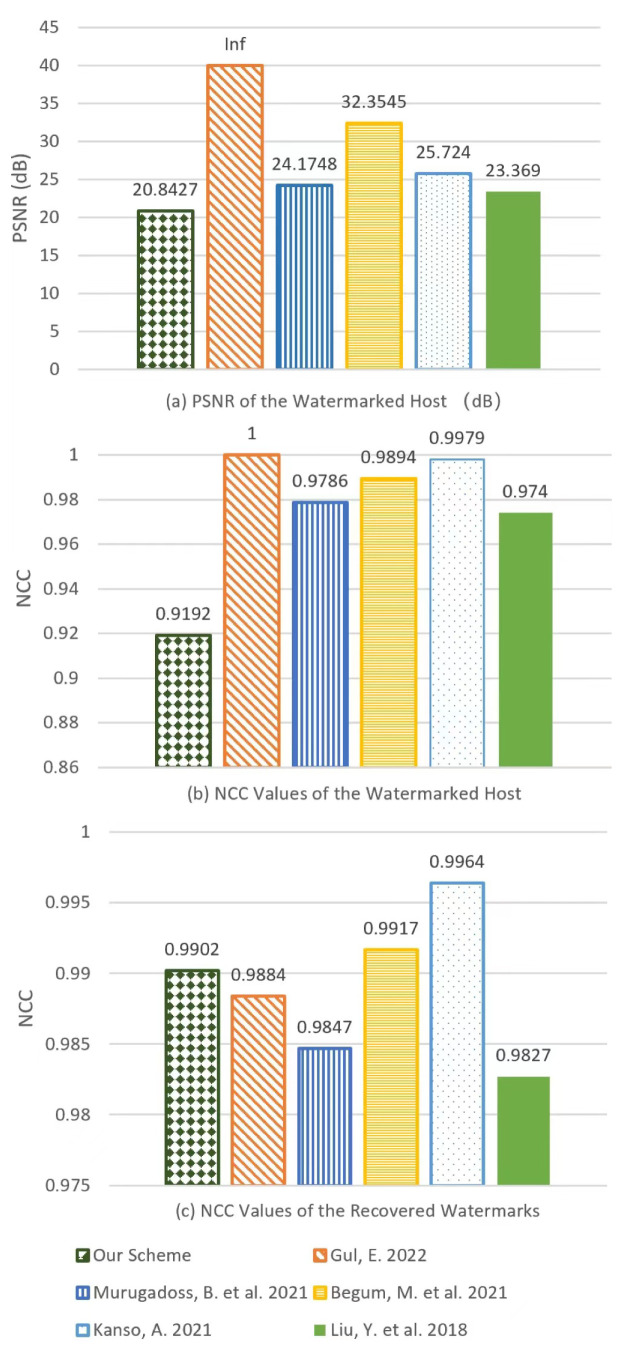
Comparison of impercipient of watermarks in different methods. (**a**) PSNR values of the host image; (**b**) NCC values of the watermarked host image; (**c**) NCC values of the recovered watermarks [18,33,34,35,36].

**Figure 14:**
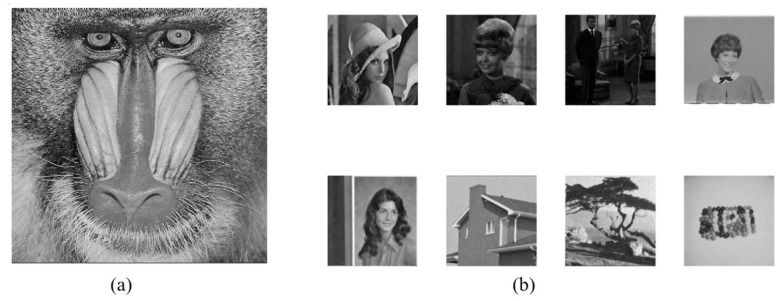
Watermarked image “baboon” and eight extracted watermarks, where no attacks involved. (**a**) watermarked host image; (**b**) recovered watermarks.

Table 2 records the PSNR and NCC values of various techniques without any attacks, which is also illustrated in Figure 13 for clarity. Although our scheme shows low indicators of imperceptibility, we consider it still to be acceptable. As shown in Figure 14, there is almost no visual distortion on the watermarked image even though eight watermarks are embedded.

### 4.4. Security

#### 4.4.1. Secret Key Sensitivity and False Positive Problem (FPP) Analysis

**Figure 15:**
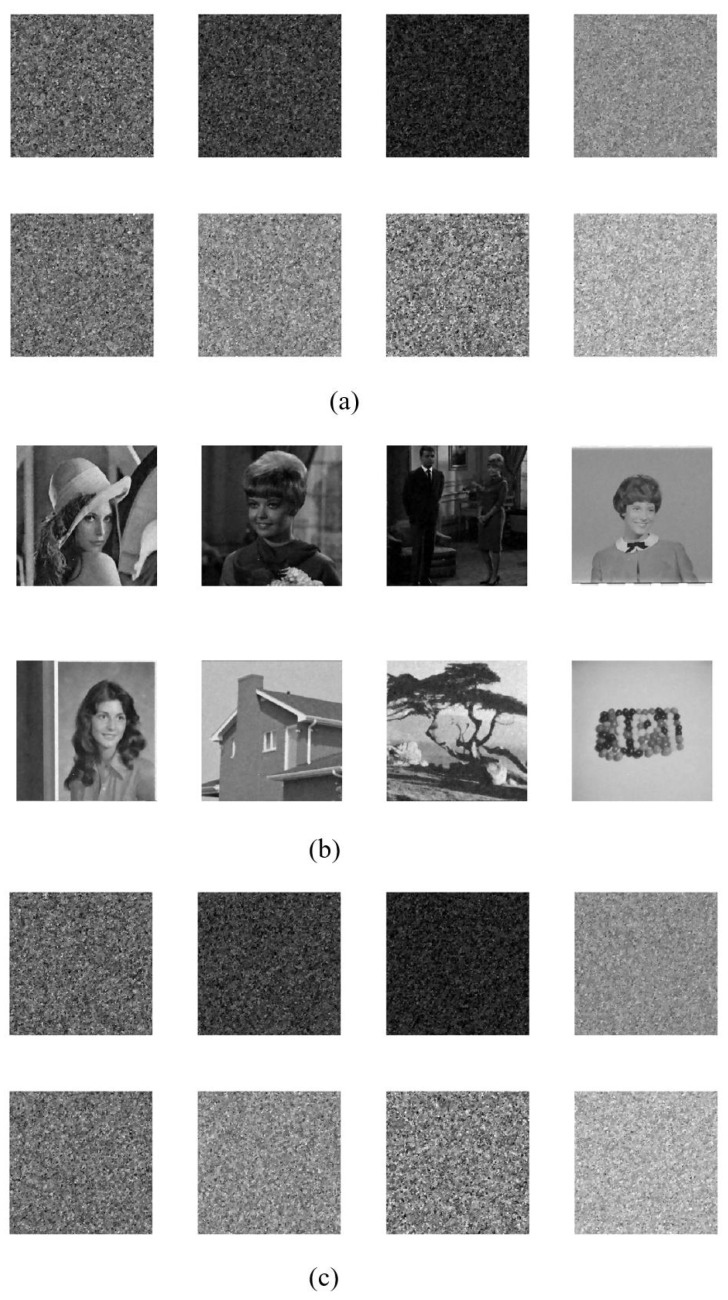
Watermarks extraction with a slight change of secret keys. (**a**) $x_{0}=0.6+\nabla$, $y_{0}$ = 0.98 (wrong), (**b**) $x_{0}$ = 0.6, $y_{0}$ = 0.98 (correct), (**c**) $x_{0}$ = 0.6, $y_{0}=0.98+\nabla$ (wrong), where $\nabla =10^{-16}$.

The application of TD-COTDCM makes our scheme very sensitive to slight variations of secret keys. As shown in Figure 15, with the correct secret key, the image can be decrypted successfully; otherwise, one can not decipher any meaningful information visually even if the secret key varies very little.

Table 3 shows different encryption mechanisms adopted in each watermarking scheme. It is known that Arnold transform has a limited transformation cycle, the image encrypted by Arnold transform can be deciphered easily by attackers with a continuous shuffling process in finite times [31]. Compared with those ordinary chaotic systems, the hyperchaotic system exploited in our scheme owns higher randomness and more complex nonlinear dynamics as well as a very large secret key space. The merits of TD-COTDCM guarantee higher security of the watermarks in our scheme than others.

Most SVD-based watermarking techniques fail to offer authentication due to their FPP [37], and many efforts were made to provide available solutions, e.g., Ganic and Eskicioglu [38] decomposed a host image with one-level DWT and then applied SVD on a grey-scale watermark image and its sub-bands; Rastegar et al. [39] proposed a hybrid SVD-based image watermarking scheme by applying Finite Radon Transform (FRT) and 3-level DWT to the host image. One-way hashing function on U and V is introduced as well in [40] for reducing FFP. However, all these techniques still suffer from FPP [37] as U and V components are stored as secret keys. Therefore, watermark encrypting becomes a valid solution since only a noise-like watermark image could be obtained if the owner could not provide the correct secret keys. In our scheme, we combine the hybrid domain transforming method and encryption method together, and thus to achieve a stronger watermark security, robustness, and higher imperceptibility. The proposed scheme is very sensitive to slight changes in the secret keys and thus is capable of overcoming the FPP [20], and serving for authentication, copyright ownership protection, and digital steganography.

#### 4.4.2. Keyspace Analysis

The TD-COTDCM has five parameters: a,b,c,d,e, which are very sensitive to boundary conditions. Its parameters have a wide range of behaving hyper-chaotically [10]. The secret key space is at least $1\times 100^{4}\times 2^{128\times 5}$, which shows its strong resistance to brute force attacks.

#### 4.4.3. Application in Color Images

**Figure 16:**
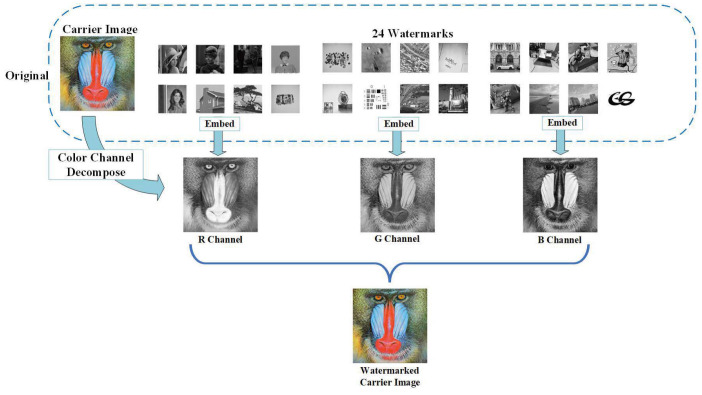
The flowchart of watermarks embedding for color image. (**a**) original color host image; (**b**–**d**) 24 grayscale watermarks.

**Figure 17:**
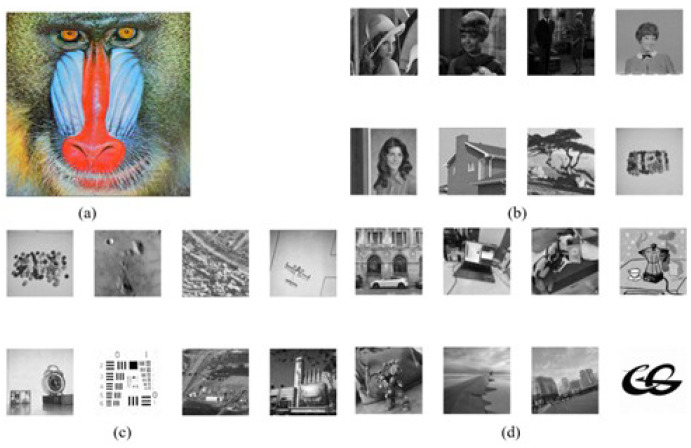
The color watermarked image “baboon” and 24 extracted watermarks. (**a**) is the host image with watermarks embedded, (**b**–**d**) separately represent 8 different watermarks inserted in the R, G, and B channels of the host image.

In addition, our method is applicable to color image watermarking as well. The processing includes decomposing the color host image into three component matrices referred to as R, G, and B channels, firstly. Each channel is deemed as a grayscale host image for watermarks embedding with the proposed scheme, subsequently. A 512×512×3 color image can be embedded in a total of 24 grayscale watermark images size 256×256. The original color image and grayscale watermarks are shown in the top part of Figure 16, while the bottom of Figure 16 illustrates the watermarked image where one could hardly perceive any visual distortions. Figure 17 displays the watermarks after decryption, which still maintain high visual quality. Indicators include PSNR and NCC values of this part are recorded in Table 4. Note that all NCC values are greater than 0.93, which exhibits a preferable performance.

## 5. Conclusions

In this paper, a robust digital watermarking scheme using CS sampling and a hyperchaos encrypting technique on a hybrid domain is proposed, which enables a 512×512 grayscale host image to accommodate up to eight grayscale watermark images with a size of 256×256. The application of CS in the proposed scheme is to maximize the utilization of capacity, which is equivalent to capacity expansion of the host image. The TD-COTDCM has a very large secret key space and ensures a measurement matrix with the property of preferable pseudo-randomness. The introduction of this hyperchaos into our scheme is another great improvement for the robust and security guarantees as well as the FPP. In addition, the execution of SVD on both watermarks and host images also benefits the capacity, robustness, and security of the proposed scheme to some extent. The experimental results show that our scheme can be effectively resistant against different levels of Gaussian noise, pepper and salt noise, JPEG compression, and cropping attacks, which reveal higher robustness than several existing methods. Although the indicators on imperception of the proposed methods are slightly lower than those of comparison methods, there is no visual distinction between the watermarked image and the host image in our scheme. Furthermore, our scheme ensures a large capacity for watermarks embedding, which applies to both grayscale and color images. It is concluded that the proposed scheme will shine in future watermarking applications.

## Figures and Tables

**Table 1:** Comparisons in NCC values under various attacks.

Attack Type	Attack Properties	Proposed Scheme	Ref. [34]	Ref. [35]	Ref. [33]	Ref. [18]	Ref. [36]
Gaussian noise	0.001	0.9899	0.9798	0.9847	0.9903	0.0035	0.9811
	0.005	0.9896	0.9587	0.9669	0.9848	0.0016	0.9582
	0.01	0.9886	0.9329	0.9464	0.9807	−0.0042	0.9296
	0.05	0.9778	0.7897	0.8679	0.9492	−0.0038	0.828
	0.1	0.9676	0.6963	0.8116	0.9264	−0.0022	0.7612
Salt & Pepper noise	0.005	0.9901	0.9816	0.9838	0.9903	0.9639	0.9781
	0.01	0.9897	0.9741	0.9751	0.9892	0.9289	0.9674
	0.05	0.9873	0.9227	0.9195	0.9788	0.7239	0.8938
	0.1	0.9829	0.8537	0.8669	0.967	0.5442	0.8326
	0.3	0.9638	0.6076	0.7356	0.9274	0.1666	0.6878
	0.5	0.9477	0.3523	0.6374	0.8943	0.0375	0.5903
Rotation	$15^{\circ }$	0.9857	0.8199	0.8791	0.9931	0.48	0.8382
	$25^{\circ }$	0.9833	0.7664	0.8217	0.9942	0.2894	0.7555
	$30^{\circ }$	0.9823	0.7397	0.8167	0.9958	0.2027	0.7409
	$45^{\circ }$	0.9807	0.721	0.8485	0.9961	0.2059	0.7744
JPEG compresison	10%	0.9901	0.9522	0.985	0.9916	0.0128	0.983
	20%	0.99	0.9277	0.9843	0.9916	−0.0009	0.9817
	30%	0.9898	0.8918	0.9807	0.9909	−0.0078	0.9772
	40%	0.9893	0.8467	0.976	0.9907	−0.0023	0.9724
	70%	0.98	0.7276	0.9577	0.9932	−0.0006	0.9552
Cropping (on the left right corner)	50×50	0.99	0.9476	0.938	0.9916	0.9794	0.9417
	100×100	0.988	0.8473	0.762	0.9938	0.931	0.7212
	150×150	0.9844	0.7203	0.647	0.9979	0.8585	0.6189
	200×200	0.9814	0.5819	0.7891	0.9996	0.7705	0.7925
	512×512	0.0038	0	−0.0034	0.9609 *	−0.0169	0

**Table 2:** PSNR and NC based comparison between various techniques without attacks.

Properties	Proposed Scheme	Ref. [34]	Ref. [35]	Ref. [33]	Ref. [18]	Ref. [36]
PSNR (dB)	20.8427	Inf	24.1748	32.3542	35.724	23.369
NCC of the embedded host	0.9192	1	0.9786	0.9894	0.9979	0.974
NCC of the recovered watermarks	0.9902	0.9884	0.9847	0.9917	0.9964	0.9827

**Table 3:** Comparison in an encryption method.

Proposed Scheme	Ref. [34]	Ref. [35]	Ref. [33]	Ref. [18]	Ref. [36]
TD-COTDCM	Arnold Transform	Henon Map	Arnold Transform	3D Cat Transform	Logistic Map

**Table 4:** PSNR and NCC values of the watermarked host image.

Properties	Embedded 8 Watermarks	Embedded 16 Watermarks	Embedded 24 Watermarks
PSNR (dB)	26.8448	23.5383	20.8481
NCC	0.9811	0.9646	0.9429

## Data Availability

Not applicable.
